# Diagnostic Utility of SARS-CoV-2 Nucleocapsid Antigenemia: A Meta-analysis

**DOI:** 10.1093/ofid/ofae561

**Published:** 2024-10-02

**Authors:** Gregory L Damhorst, Sydney E Martin, Eli Wilber, Hans Verkerke, Michael Goodman, Wilbur A Lam

**Affiliations:** Division of Infectious Diseases, Department of Medicine, Emory University, Atlanta, Georgia, USA; The Atlanta Center for Microsystems-Engineered Point-of-Care Technologies, Atlanta, Georgia, USA; Department of Pediatrics, Emory University School of Medicine, Atlanta, Georgia, USA; Division of Infectious Diseases, Department of Medicine, Emory University, Atlanta, Georgia, USA; Emory University School of Medicine, Atlanta, Georgia, USA; Rollins School of Public Health, Emory University, Atlanta, Georgia, USA; The Atlanta Center for Microsystems-Engineered Point-of-Care Technologies, Atlanta, Georgia, USA; Department of Pediatrics, Emory University School of Medicine, Atlanta, Georgia, USA; Aflac Cancer & Blood Disorders Center at Children's Healthcare of Atlanta, Atlanta, Georgia, USA; Wallace H. Coulter Department of Biomedical Engineering, Georgia Institute of Technology, Atlanta, Georgia, USA

**Keywords:** antigenemia, nucleocapsid, SARS-CoV-2

## Abstract

**Background:**

Studies of the diagnostic performance of severe acute respiratory syndrome coronavirus 2 (SARS-CoV-2) nucleocapsid antigen in blood (antigenemia) have reached variable conclusions. The potential utility of antigenemia measurements as a clinical diagnostic test needs clarification.

**Methods:**

We performed a systematic review of Pubmed, Embase, and Scopus through July 15, 2023, and requested source data from corresponding authors.

**Results:**

Summary sensitivity from 16 studies (4543 cases) sampled at ≤14 days of symptoms was 0.83 (0.75–0.89), and specificity was 0.98 (0.87–1.00) from 6 studies (792 reverse transcription polymerase chain reaction–negative controls). Summary sensitivity and specificity for paired respiratory specimens with cycle threshold values ≤33 were 0.91 (0.85–0.95) and 0.56 (0.39–0.73) from 10 studies (612 individuals). Source data from 1779 cases reveal that >70% have antigenemia 2 weeks following symptom onset, which persists in <10% at 28 days. The available studies suffer from heterogeneity, and Omicron-era data are scarce.

**Conclusions:**

Nucleocapsid antigenemia currently has limited utility due to limitations of existing studies and lack of Omicron-era data. Improved study designs targeting potential clinical uses in screening, surveillance, and complex clinical decision-making—especially in immunocompromised patients—are needed.

Coronavirus nucleocapsid protein was first observed in the blood of individuals with severe acute respiratory syndrome (SARS) during the 2003 outbreak in Guangzhou, China [1–5]. The SARS coronavirus 2 (SARS-CoV-2) nucleocapsid protein is a 45.6-kDa, 419–amino acid protein coded near the 3′ end of the viral genome, is important to virus structure and replication, and exhibits high similarity to the SARS-CoV nucleocapsid protein [6–8]. The scientific community's response to the global pandemic of SARS-CoV-2 beginning in 2019 generated unprecedented quantities of diagnostic test–related data. Numerous studies have described quantitative viral nucleocapsid protein measurements in serum or plasma (antigenemia) framed as a marker of acute infection or a predictor of disease severity [9]. Yet a defined role for antigenemia measurements in clinical care or public health remains unclear.

The appeal of a quantitative blood-based biomarker for SARS-CoV-2 infection includes the following: (1) a sample type less subject to variability than technique-dependent respiratory specimens, (2) possible utility in surveillance utilizing already collected blood specimens when respiratory sampling is burdensome or not available, and (3) potential to reflect disease processes in ways not captured by an upper respiratory swab specimen, such as compartmentalized infection or disease monitoring in immunocompromised hosts. We sought to assess the quality and quantity of evidence for nucleocapsid antigenemia as a diagnostic biomarker through a systematic review of all available published data. We requested source data from corresponding authors of each study and extracted results from publications when necessary to perform meta-analyses of sensitivity and specificity with respect to clinically defined acute coronavirus disease 2019 (COVID-19) and paired respiratory reverse transcription polymerase chain reaction (RT-PCR) cycle threshold (Ct) values ≤33. We also performed an aggregate data analysis to estimate antigenemia kinetics. Our analysis highlights the need for precisely designed studies of antigenemia with modern variants to validate potential clinical uses.

## METHODS

### Search Strategy

A search of Pubmed, Embase, and Scopus was performed on February 25, 2023 (updated July 15, 2023), using terms for SARS-CoV-2 infection (“COVID,” “SARS-CoV-2,” or “coronavirus”), the nucleocapsid biomarker (“nucleocapsid,” “N protein,” or “antigen”), and sample type (“blood,” “plasma,” or “serum”) or the term “antigenemia” (see Supplementary Tables 1–3 for full search terms). Results were initially imported into EndNote 20 to update and standardize metadata and remove duplicate records.

### Systematic Review

De-duplicated publication records were imported into the COVIDence web application (Melbourne, Australia). Titles and abstracts were screened for publications describing nucleocapsid antigenemia measurements. Full texts were then assessed for baseline inclusion criteria: (1) quantitative viral nucleocapsid measurements in serum or plasma expressed in terms of mass per unit volume and (2) cases defined by positive SARS-CoV-2 testing. At both stages, studies excluded by both authors were omitted from further review and studies included by at least 1 author were passed to the next stage. Reasons for exclusion are provided in Supplementary Figure 1. The protocol for this review was not prospectively registered, and the protocol was not final before beginning data extraction.

### Data Extraction

Data independently extracted by 2 authors included study date, location, assay platform, cutoff value, case definition, cases and controls with and without antigenemia, and subsets of these within 7 or 14 days since symptom onset and with paired respiratory RT-PCR Ct values ≤ or >33. Conflicts between the 2 authors were discussed to reach consensus.

The corresponding authors of all included studies were contacted on or after May 9, 2023, with a request for original data including duration of symptoms at sample collection, month, year, patient age, precise antigenemia level, and concurrent nasal swab cycle threshold (Ct) value. The authors of 17 studies provided data (Table 1) [10–15, 17–39]. Source data were used to recalculate true positives (TPs), false positives (FPs), true negatives (TNs), and false negatives (FNs) using an index test cutoff of 2.97 pg/mL and to analyze antigenemia kinetics.

**Table 1.** Characteristics of Included Studies With Data Included in a Meta-analysis

First Author	Ref	Pub Year	Location	Start Date	End Date	Ages (Cases)	Assay	Cutoff, pg/mL	Source Data	Population	Definition of Case	Definition of Control
Ahava	[10]	2022	Helsinki, Finland	3/1/2020	5/31/2020	Me 54	Salocor Antigen Quantitative Assay Kit	2.97	No	Samples sent to hospital laboratory	RT-PCR+	NA
						R 24–86						
Blain	[11]	2022	Montpellier, France	3/1/2021	4/30/2021	Me 91.1	COV-QUANTO	2.97	Yes	Nursing home residents	NP RT-PCR+	NA
						R 74.7–101.8						
Chenane	[12]	2022	Paris, France	1/25/2020	9/2/2020	Me 63	COV-QUANTO	2.98	No	French COVID cohort	See clinical trial	NA
						IQR 52–71						
Damhorst	[13]	2023	Atlanta, Georgia, USA	3/17/2020	3/30/2021	Me 14	Quanterix Simoa	0.099	Yes	Hospitalized children	−3 to +14 d since RT-PCR+ and and within ≤14 d of symptoms; ≤10 d of hospitalization	NA
						R 0–20						
Damhorst	[14]	2023	Atlanta, Georgia, USA	1/4/2022	2/16/2022	Me 54.5	Quanterix Simoa	3	Yes	Inpatient or emergency department	RT-PCR+	NA
						IQR 46–68						
Favresse	[15, 16]	2022	Belgium	NR	NR	Me 77	Quanterix Simoa	0.099	Yes	Inpatient and outpatient	RT-PCR+ within 1 d^a,b^	NA
						R 20–97						
Hingrat	[17]	2020	France	1/25/2020	9/2/2020	NR	COVID-19 Quantigene CE-IVD ELISA microplate assays	2.98	No	Clinical trial cohorts French COVID (NCT04262921) and CoV-CONTACT (NCT04259892)	See clinical trials	NA
Jilg	[18]	2023	Multinational	1/1/2021	7/31/2021	NR	Quanterix Simoa	3	No	Outpatient (ACTIV-2 trial)	See clinical trial	NA
Li	[19]	2020	Anhui Province, China	NR	NR	NR	BIOHIT ELISA	2	No	Inpatient	RT-PCR+ and CT changes	RT-PCR & N Ab negative
Ogata	[20]	2020	Boston, Massachusetts, USA	3/25/2020	5/20/2020	Me 63	Quanterix Simoa	0.02	Yes	Patients presenting to hospital	RT-PCR+	RT-PCR-
						R 19–91						
Oueslati	[21]	2022	France	3/1/2020	12/31/2020	NR	COV-QUANTO	2.98	No	Patients with respiratory symptoms	RT-PCR+ same day	RT-PCR-
Parraud	[22]	2023	France	3/1/2020	4/30/2020	Me 67	COV-QUANTO	3	Yes	Inpatient	RT-PCR+	NA
						R 27–86						
Perna	[23]	2021	Italy	NR	NR	Mn 60	Lumipulse G SARS-CoV-2 Ag CLEIA	0.1^c^	Yes	Inpatient	COVID-19	NA
						SEM 7						
Rogers	[24]	2022	Multinational	8/1/2020	11/15/2021	Me 57	Quanterix Simoa	3	No	TICO clinical trial (inpatients)	≤12 d of symptoms	NA
						IQR 46–68						
Saini	[25]	2023	Chicago, Illinois, USA	NR	NR	Me 35	Quanterix Simoa	1.25	Yes	Outpatient and inpatient		SC2 negative
						R 27–67						
Shan	[26]	2021	Bonn, Germany	3/30/2020	6/17/2020	NR	Quanterix Simoa	1.25	SI	Inpatient and commercial source	RT-PCR+	NA
Sigal	[27]	2022	USA	6/17/2020	6/17/2021	R 0.1–20.8	MSD S-PLEX	1.28	Yes	Overcoming COVID-19 Immunobiology Study (inpatients)	RT-PCR+ same day and symptoms	RT-PCR- same day
Su	[28]	2021	Beijing, China	NR	NR	Mn 57.9	Quanterix Simoa	0.0157	No	Hospital, inpatient or outpatient not specified	RT-PCR+	RT-PCR-
						SD 15.1						
Sullivan	[29]	2023	USA	8/1/2020	7/31/2021	NR	Quanterix Simoa	3	No	Inpatient	RT-PCR+ within 14 d	NA
Swank	[30]	2022	Boston, Massachusetts, USA	NR	NR	Me 53	Quanterix Simoa	NR	Yes	COVID-19 with and without PASC	Previously infected with SARS-CoV-2^a^	NA
						R 0–83						
Thudium	[31]	2021	Denmark	3/3/2020	2/2/2021	Me 50.3	Solsten ELISA^d^	10	Yes	Inpatient and outpatient	Upper respiratory tract PCR+^b^	URT PCR negative
						R 13.9–101.8						
Verkerke	[32]	2021	Atlanta, Georgia, USA	NR	NR	NR	Quanterix Simoa	0.099	Yes	Inpatient	RT-PCR+ with symptom onset documented^a^	NA
Verkerke	[33]	2022	Atlanta, Georgia, USA	1/11/2021	3/12/2021	Mn 60.6	Quanterix Simoa	0.099	Yes	Inpatient and outpatient	SC2+ test (any) and −3 to 14 d since symptom onset	RT-PCR- same day
						IQR 52.2–73.0						
Veyrenche	[34]	2022	Montpellier, France	3/1/2020	5/31/2021	Me 63.0	COV-QUANTO	2.97	Yes	Inpatient	SC2 infected	NA
						R 30.0–98.5						
Wang	[35]	2021	San Francisco, California, USA	3/1/2020	11/30/2020	Me 56	MSD S-PLEX	0.631	Yes	Inpatient and outpatient	NAAT+ and symptomatic	NAAT-
						R 0–94						
Wick	[36]	2022	San Francisco, California, USA	3/1/2020	8/31/2021	Me 54.7	Quanterix Simoa	3	Yes	Inpatient	RT-PCR+	NA
						R 18.6–88.2						
Yonker	[37]	2021	Boston, Massachusetts, USA	NR	NR	Me 14	Quanterix Simoa	1.45	No	Inpatient and outpatient	RT-PCR+	NA
						R 0.04–22						
Zhang	[38]	2021	San Francisco, California, USA	3/1/2020	7/31/2020	Me 48	BIOHIT	2.97	No	Inpatient and outpatient	RT-PCR+	RT-PCR-
Zhang	[39]	2022	Anhui Province, China	1/1/2020	2/28/2020	Me 49 (Ag+)	BIOHIT ELISA	10	No	Inpatient	RT-PCR+	NA
						Me 31 (Ag-)						

Abbreviations: COVID-19, coronavirus disease 2019; CT, computed tomography; ELISA, enzyme-linked immunosorbent assay; IQR, interquartile range; Me, median; Mn, mean; NA, not applicable; NAAT, nucleic acid amplification test; NP, nasopharyngeal; NR, not reported; PASC, postacute sequelae of SARS-CoV-2 infection; R, range; RT-PCR, reverse transcription polymerase chain reaction; SARS-CoV-2, severe acute respiratory syndrome coronavirus 2; SC2, SARS-CoV-2; SI, source data in supplementary information.

^a^For studies with a significant number of measurements >2 weeks after COVID-19 symptom onset, only those with ≤14 days of symptoms were included in the least restrictive meta-analysis.

^b^For studies with a significant number of measurements >2 weeks after first SARS-CoV-2-positive test, only those ≤14 days since positive testing were included in the least restrictive meta-analysis.

^c^Cutoff value for Perna et al. was determined from source data.

^d^The CE-IVD labeled, Solsten SARS-CoV-2 Antigen ELISA Kit (Solsten Diagnostics Intl., Aarhus, Denmark).

### Reference Standards

Acute COVID-19 (defined by RT-PCR respiratory testing and clinical parameters) and nasal swab RT-PCR Ct values were considered as references standards in separate meta-analyses (Supplementary Figure 2).

### Index Test

The index test was a quantitative viral nucleocapsid protein level in plasma or serum (antigenemia). The most frequently used cutoff across studies fell within a narrow range (2.97–3.0 pg/mL) (Table 1). The lower limit of this range was chosen as a standardized cutoff, and only studies where positive antigenemia was defined by a cutoff value of 2.97, 2.98, or 3 pg/mL or where source data were provided such that diagnostic performance could be recalculated using the 2.97-pg/mL cutoff were included in the primary meta-analysis. Alternate analyses that allow for heterogenous cutoff values are provided in Supplementary Figures 3 and 4.

### Acute COVID-19 Meta-analysis

We performed 2 alternate meta-analyses with respect to acute COVID-19, which was inconsistently defined across studies. First, the authors’ definition of acute COVID-19 was accepted. Alternately, only specimens obtained from patients with positive SARS-CoV-2 respiratory testing and ≤14 days of symptoms were included. An additional alternate analysis of cases with ≤7 days of symptoms is in Supplementary Figure 3.

Multiple measurements from the same patient were included in the meta-analysis if they originated from different time points during the disease process. Parameters were placed on source data, including when the reference was the authors’ definition of acute COVID-19 if serial measurements from acute COVID-19 cases were provided for weeks or months after diagnosis and/or symptom onset (Supplementary Table 4). For the meta-analysis limited to cases with ≤14 days of symptoms, TP and FN were extracted when symptom duration could be determined. When grouped based on symptom duration, all quanta not exceeding 14 days of symptoms were included.

A meta-analysis of specificity was performed using measurements from patients with negative SARS-CoV-2 testing. No specific parameters on timing of the negative testing were applied, and many studies did not provide this information. Prepandemic specimens were not included in the specificity meta-analysis.

The data elements for the meta-analyses included TP, FP, TN, and FN observations from source data or data extracted from the published manuscript and supplementary data. If not explicitly stated, TP and FN were calculated from the reported frequency (f) of antigenemia multiplied by the total number of cases (N) and rounding the result to the nearest integer (TP = f × N, FN = N – TP). Outputs of the meta-analyses were pooled sensitivity and specificity estimates and 95% confidence intervals. Each analysis included a test for heterogeneity expressed as an *I*^2^ estimate, and a *P* value was derived from the Q test as well as the chi-square test for differences in subgroups based on common assay platforms (Quanterix Simoa, COV-QUANTO, or “other assays” to encompass all assays not appearing >3 times in the included literature). Meta-analyses were performed in R using the packages *meta* and *mada* and the function *metaprop* [40]. The sample sizes of cases and controls used in primary and alternate meta-analyses are summarized in Supplementary Table 5.

### Viral Load (Ct ≤33) Meta-analysis

The second reference standard applied only to the subset of patients with concurrent SARS-CoV-2 RNA detected in a nasopharyngeal specimen where a Ct value was provided. Ct values are inversely related to quantity of viral RNA and are commonly interpreted as a surrogate marker for active viral replication and/or infectivity. Only studies reporting Ct values and nucleocapsid antigenemia measurements performed from specimens collected within 24 hours were included. Analysis was not restricted by viral gene target used by the RT-PCR. The reference standard for high viral load was Ct value ≤33, as utilized in several studies [14, 16, 17, 21].

A viral load meta-analysis was also performed in R. As the patients in the studies included in this meta-analysis met uniform criteria (RT-PCR+), a summary ROC curve was produced using the *mada* package and the *retisma* function.

### Risk of Bias and Applicability Assessment

The Quality Assessment of Diagnostic Accuracy Studies (QUADAS-2) tool was used as a framework for assessing risk of bias and applicability of each study (Supplementary Table 6) [41]. A single reviewer (G.L.D.) performed the assessment. Because separate meta-analyses were performed for 2 different reference standards, separate bias assessments were performed.

### Role of the Funding Source

This work was supported by the National Institute of Biomedical Imaging and Bioengineering of the National Institutes of Health (award number U54EB027690) as part of the Rapid Acceleration of Diagnostics (RADx) initiative. The funding source had no role in the design, implementation, or interpretation of the study.

## RESULTS

### Systematic Review and Data Extraction

A total of 7418 records were retrieved from our search. After removal of duplicates, 3686 titles with abstracts were screened (Supplementary Figure 1). Full texts of 146 studies were reviewed, and 44 studies met the inclusion criteria [10–15, 17–39, 42–54]. The authors of 17 studies provided source data [11, 13–15, 20, 22, 23, 25–27, 30–36], 13 studies presented sufficient detail for their results to be included in the main and/or supplementary meta-analysis (Table 1) [10, 12, 17–19, 21, 22, 24, 28, 29, 37–39], and 14 studies did not present data in sufficient detail to be included (Supplementary Table 7) [42–54].

### Sensitivity and Specificity for Acute COVID-19

**Figure 1.**
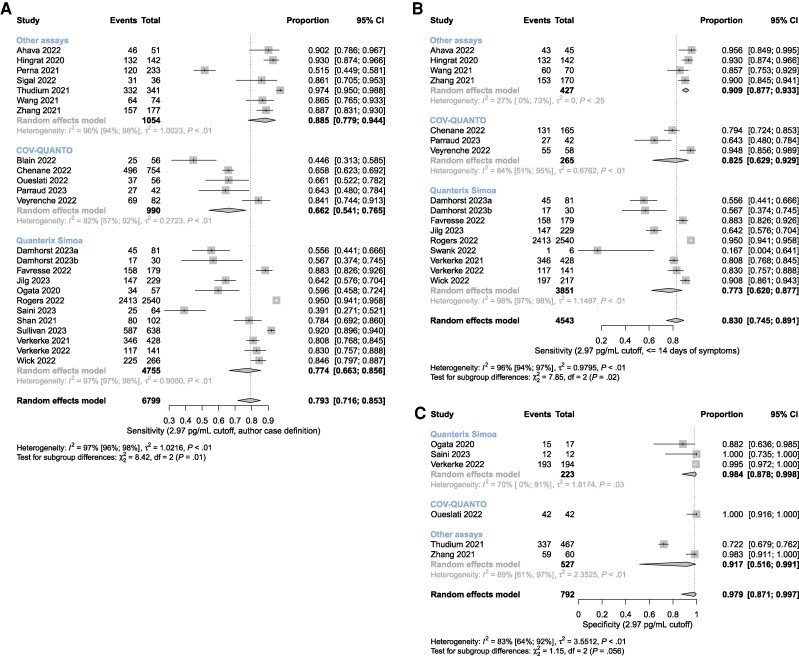
Meta-analyses of nucleocapsid antigenemia as a diagnostic marker of acute COVID-19 based on clinical diagnosis using an index text cutoff of 2.97 pg/mL. Studies are grouped according to the assay used (Quanterix Simoa, COV-QUANTO, and other assays). A, Sensitivity using the authors’ definition of acute COVID-19. B, Sensitivity only for those cases with ≤14 days of symptoms. C, Specificity for SARS-CoV-2-negative individuals. Abbreviations: COVID-19, coronavirus disease 2019; SARS-CoV-2, severe acute respiratory syndrome coronavirus 2.

Summary sensitivity using the authors’ case definition was 0.79 (95% CI, 0.72–0.85) with significant heterogeneity (*I*^2^ = 97% [96%–98%]) (Figure 1A). Summary sensitivity for cases within 14 days of symptom onset was 0.83 (0.75–0.89) and exhibited similar heterogeneity (*I*^2^ = 96% [94%–97%]) (Figure 1B). Summary specificity for SARS-CoV-2-negative persons was 0.98 (0.87–1.00) with an *I*^2^ of 83% (64%–92%) (Figure 1C).

### Sensitivity and Specificity of Antigenemia for Ct Value ≤33

**Figure 2.**
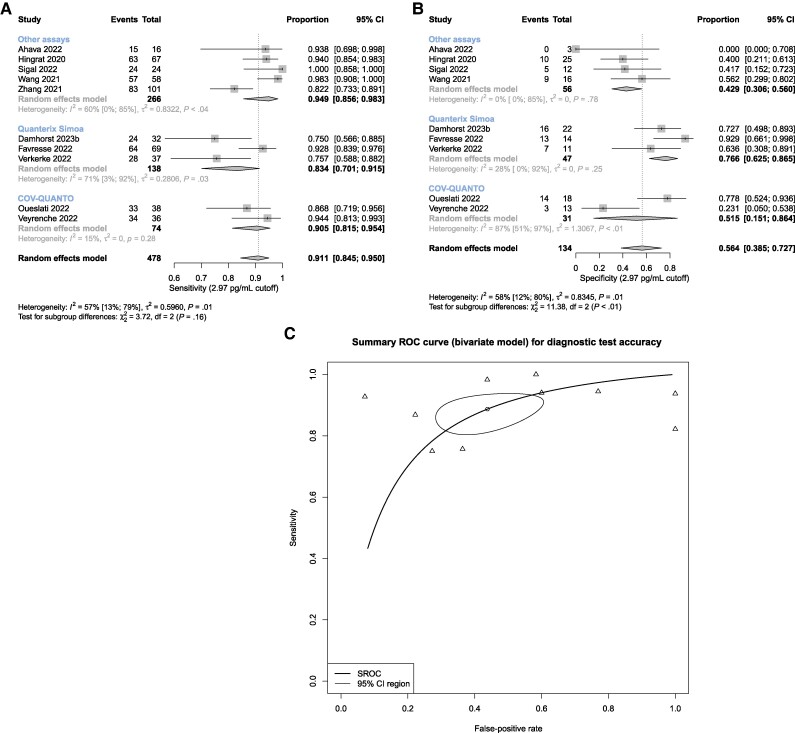
Meta-analysis of nucleocapsid antigenemia as a diagnostic indicator of nasal swab RT-PCR Ct value ≤33. Univariate models for (A) sensitivity and (B) specificity, and (C) summary ROC curve using a bivariate model. Abbreviations: Ct, cycle threshold; ROC, receiver operating characteristics; RT-PCR, reverse transcription polymerase chain reaction.

Summary sensitivity for antigenemia with respect to a respiratory RT-PCR Ct value ≤33 was 0.91 (0.85–0.95) with an *I*^2^ of 57% (13%–79%) (Figure 2A). Summary specificity was 0.56 (0.39–0.73; *I*^2^ = 58% [12%–80%]) (Figure 2B). Summary area under the curve of the bivariate ROC model was 0.82 (Figure 2C). The gene targets included in each study are summarized in Supplementary Table 8.

### Assay Subgroup Analyses

Across-group differences were statistically significant in the sensitivity meta-analyses utilizing the authors’ definition and symptoms ≤14 days to define acute COVID-19 (*P* = .01 and < .01, respectively) (Figure 1) and in the specificity meta-analysis for high concurrent upper respiratory tract Ct value (*P* < .01) (Figure 2). However, the within-group heterogeneity was still pronounced, and even instances where *I*^2^ values were relatively small (eg, <40%) the apparent homogeneity of assay-specific results was likely attributable to the relatively small number of studies with wide, largely overlapping confidence intervals. To further clarify effects of outliers in light of high heterogeneity, a sensitivity analysis for assay subgroups with *I*^2^ >90% is provided in Supplementary Table 9, which does not reveal any substantial influence of outliers on overall sensitivity estimates.

### Timing of Studies

**Figure 3.**
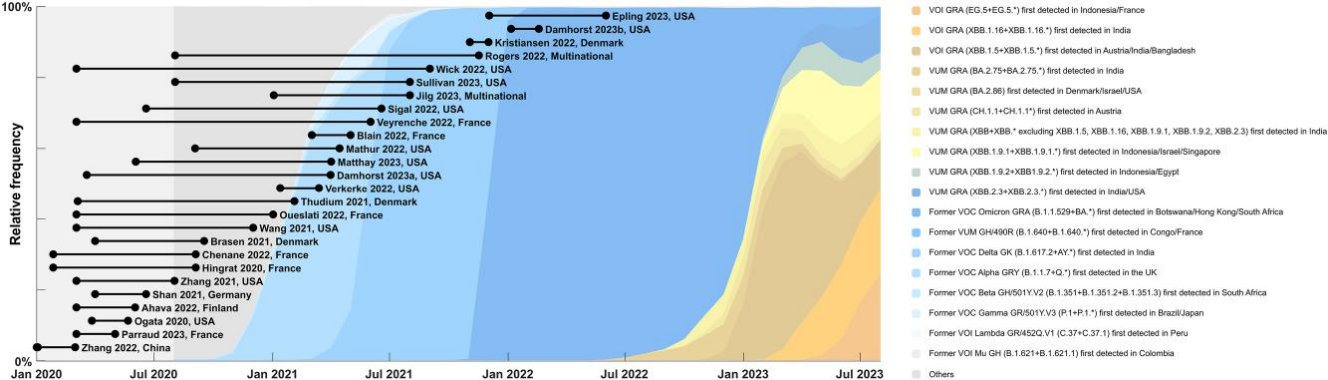
Timeline of studies identified in our review superimposed on variant trends retrieved from GISAID.org. The relative frequency of each variant is depicted as a fraction of the sequenced isolates. Studies that did not indicate specimen collection dates were omitted.

No study systematically evaluated differences in antigenemia between major variants. Only 2 studies collected data during the Omicron era (Figure 3).

### Kinetics of Antigenemia

**Figure 4.**
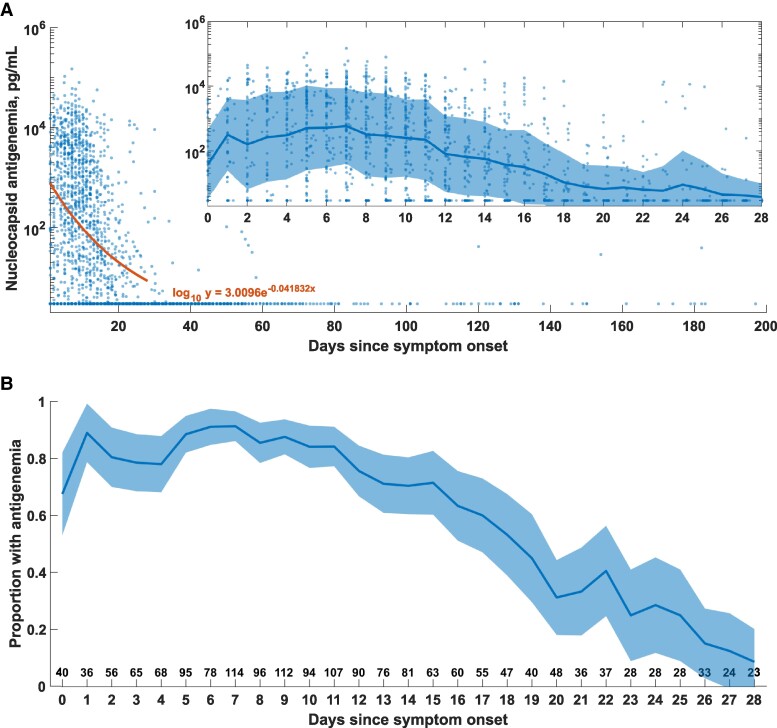
Antigenemia kinetics. A, Antigenemia levels vs days since symptom onset for source data from 10 studies. All measurements below the threshold value were plotted at 2.97 pg/mL. An exponential fit to the log_10_ of antigenemia level vs day of symptoms was performed. A-inset axes, Details of 28-day kinetics following COVID-19 symptom onset. The line and shaded region represent the mean and standard deviation of the log_10_ of the antigenemia level. B, Proportion of patients represented in source data with antigenemia >2.97 pg/mL as a function of days of symptoms. For data where more precise symptom durations were provided, values were rounded down to the nearest integer. The shaded region represents the 95% CI calculated for each time point using the formula for standard error. The number below indicates the total number of cases in the aggregate data set at each time point. Abbreviation: COVID-19, coronavirus disease 2019.

Source data with symptom onset recorded were available for 1779 patients from 9 studies [13–15, 30, 32–36]. Most antigenemia was observed in the first 4 weeks following symptom onset, and levels waned and nearly disappeared by 28 days (Figure 4A). More than 70% of patients had antigenemia after 2 weeks (Figure 4B), but these data are likely biased toward hospitalized patients with more severe disease.

### Quality Assessment

Bias assessments for each included study are presented in Supplementary Table 10.

## DISCUSSION

### Interpretation of Meta-analyses

The clinical role of SARS-CoV-2 nucleocapsid antigenemia measurements is unclear. Most potential applications would rely on adequate diagnostic performance with respect to acute COVID-19 or high respiratory viral load. Our meta-analysis suggests that antigenemia is a moderately sensitive marker of acute COVID-19 (0.83 [0.75–0.89]) and a highly sensitive (0.91 [0.85–0.95]) but nonspecific (0.56 [0.39–0.73]) marker of high respiratory tract viral loads. Our conclusions are limited by between-study heterogeneity and lack of modern variant data.

A significant source of heterogeneity between studies is the definition of acute COVID-19. Using the authors’ definition of acute COVID-19 as the reference standard showed lower summary sensitivity (0.793) than when the reference standard was restricted to cases within 14 days of symptom onset (sensitivity, 0.830) (Figure 1). Many acute COVID-19 antigenemia studies may therefore be at risk of inappropriately including recovered patients with persistent detectable RNA who present to care with illness not due to SARS-CoV-2 infection.

A prior systematic review that did not perform a meta-analysis concluded that timing of sample collection, assay platform, and disease severity influence antigenemia measurements [9]. We have addressed timing to the extent possible with a reference standard based on symptom onset. Difference in severity remains an important source of between-study heterogeneity. We were not able to adjust the analysis for differences in severity, nor did we attempt a meta-analysis of the prognostic value of antigenemia because of the lack of standardized and granular severity data across studies. Several studies have independently demonstrated an association of antigenemia levels with severity of SARS-CoV-2 infection [15, 24, 33, 36]. Meanwhile, we do observe that acute COVID-19 with absent or short-lived antigenemia seems plausible, and we would expect this to be more common in less severe cases. Two antigenemia studies with rigorously selected cohorts, the ACTIV-3/TICO cohort (hospitalized cohort, 95% antigenemia) [24] and the ACTIV-2 (nonhospitalized cohort, 64% antigenemia) [18], illustrate this conclusion.

Heterogeneity can also be attributed to the use of different assay platforms, which included both ultrasensitive and standard immunoassays. Forest plots in this analysis include subgroups based on the 2 most common assays, Quanterix Simoa and COV-QUANTO, while all other assay platforms were used less frequently so they were categorized into a third subgroup, “other assays.” Although across-group differences were statistically significant in some analyses, significant within-group heterogeneity was still observed in most subgroups. The overall small number of studies and wide overlapping confidence intervals limit conclusions from these observations, but nonetheless highlight needs for validation and clarification of unique cutoff values for each assay platform and clinical application.

Sampling RT-PCR-negative individuals results in a high estimate of specificity but likely overestimates true specificity due to limited challenge bias [55]. More accurate estimates of specificity should come from studies of diagnostic performance with consecutive or random enrollment rather than case–control designs.

RNA persistence following resolution of acute COVID-19 is well documented, motivating interest in using antigenemia to adjudicate resolved active infection [56]. Comparison of antigenemia with nasal swab RT-PCR Ct value, a surrogate of respiratory tract viral load, suggests high sensitivity but poor specificity. Limitations to the interpretation of these data must also be acknowledged: Ct values do not necessarily correlate across instruments or laboratories [57], many studies present data from multiple RT-PCR instruments, and different viral gene targets are included in the meta-analyzed data. Further, many are qualitative assays and are not validated for quantitative RT-PCR.

Use of respiratory tract biomarkers to assess active infection is additionally flawed, as discordance between nasal swab findings and lower respiratory tract findings has been described [58–60]. While Ct value is often used as a surrogate for active infection and transmission potential, more labor-intensive viral culture assays may be a better reference standard. Only 2 studies compare antigenemia with viral culture and report somewhat promising findings that absence of antigenemia may help rule out a contagious state, but in total these are inconclusive owing to small sample sizes [14, 48].

The rapid evolution of SARS-CoV-2 as described by epidemiologically relevant variants and subvariants as well as current widespread immunity due to prior natural infection and vaccination may limit the applicability of the current literature, as the most recent data come from early 2022, shortly after the emergence of the Omicron variant (Figure 3). The N gene has been relatively conserved across variants compared with spike antigen, and a limited analysis of Omicron compared with early pandemic antigenemia suggests that similar ranges are observed (Supplementary Figure 5). Eighteen of 44 studies (including 9 of 30 studies included in our meta-analyses) did not clarify the dates that sampling was performed, and among the 26 studies with dates reported (including studies identified without analyzable data), only 2 likely capture the Omicron variant (Figure 3). Further, preexisting immunity due to vaccination or prior infection—which has progressed dramatically since the emergence of SARS-CoV-2—likely exerts a greater influence on antigenemia than inherent viral genomic changes. Granular vaccination and prior infection data were not provided in most publications, and we were unable to perform an analysis to further evaluate the impact of preexisting immunity on antigenemia. This will be an important area of future study.

### Antigenemia Kinetics

Before this review, duration of antigenemia was uncertain. Using available source data, which we expect are biased toward hospitalized patients, we performed an aggregate analysis of antigenemia kinetics relative to symptom onset (Figure 4). Our analysis suggests that antigenemia persists in >70% of hospitalized patients for ∼2 weeks after symptom onset and wanes by the end of the fourth week in >90% of patients. This establishes the third and fourth weeks after symptom onset as a period of uncertainty in the interpretation of antigenemia measurements. We do not have sufficient data to describe how many of these cases were still symptomatic or who may have a clinical picture consistent with persistent (or protracted) SARS-CoV-2 infection [61, 62].

### Clinical Scenarios for Future Investigation

Considering the available data, our opinion is that nucleocapsid antigenemia measurements cannot serve as an alternative to RT-PCR in patients presenting for evaluation of COVID-19-like symptoms. This stems from imperfect sensitivity (estimated through meta-analysis to be 79.3%) in cohorts with presumptive acute COVID-19. However, clinical scenarios should be considered where antigenemia measurements provide value but still require validation.

The most promising role for antigenemia is in evaluation of complex clinical presentations in immunocompromised persons who are at risk for complications of SARS-CoV-2 infection including persistent infection [58, 61–63]. Immunocompromised patients may present with syndromes where persistent SARS-CoV-2 infection is considered among other diagnoses and often require extensive workup including advanced imaging and bronchoscopy. In some of these cases, discordant testing has been observed with RT-PCR-negative nasal swabs but compelling evidence of active infection in the lower respiratory tract (authors’ unpublished observations) [58–60]. A multidisciplinary evaluation of such cases often involves consideration of biomarkers for atypical bacteria, endemic fungi, and mold infections that have poor performance characteristics [64–68]. Our aggregate analysis of antigenemia kinetics (Figure 4) raises the hypothesis that persistent antigenemia beyond 4 weeks may support a diagnosis of persistent SARS-CoV-2 infection. Prospective studies to determine duration of antigenemia in immunocompromised individuals and correlation with symptoms and viral and immune biomarkers are needed. Antigenemia to guide therapy such as duration of antivirals or administration of convalescent plasma in these patients should also be investigated.

Another application may be surveillance in inpatient health care settings where universal nasal swab sampling is not performed but blood sampling is performed. While false-positive antigenemia screening should be expected in some patients with resolved SARS-CoV-2 infection presenting during weeks 3–4 following symptom onset, universal testing of blood samples collected for routine testing may still provide an estimate of infection prevalence.

Three papers examined antigenemia in the SARS-CoV-2-associated multisystem inflammatory syndrome in children (MIS-C), but with conflicting findings [13, 27, 37]. Nucleocapsid antigenemia was identified in 9 of 16 children (56.3%) with MIS-C by Yonker et al. [37] but only 3 of 53 (5.7%) in the study Sigal et al. [27], and our prior study did not find nucleocapsid antigenemia in any of 26 MIS-C cases [13]. Notably, time since COVID-19 or SARS-CoV-2 exposure was a median (range) of 26 (12–62) days in the Yonker et al. study, and 2 of 3 antigenemic patients in the Sigal study had a recent positive SARS-CoV-2 RT-PCR, indicating that these patients may have had waning antigenemia from the primary infection. Viral spike antigenemia, which is beyond the scope of this review, was more heavily associated with MIS-C cases in the study by Yonker et al. but still rare in the other 2 MIS-C cohorts.

Limited study of the broadly defined postacute sequelae of SARS-CoV-2 infection (PASC) has also been performed, finding persistent nucleocapsid antigenemia in 1 of 12 patients for months after COVID diagnosis [30]. Further study of MIS-C, PASC, and other SARS-CoV-2-associated sequelae is needed and may warrant comprehensive assessment of viral components in a range of sample types.

Future studies aiming to characterize antigenemia as a biomarker or evaluate diagnostic performance should provide dates of sample collection and/or variant status of cases, time of sample collection relative to symptom onset, and immune status of the patients including reasons for immune compromise, vaccination status, and history of prior SARS-CoV-2 infections.

## CONCLUSIONS

SARS-CoV-2 nucleocapsid antigenemia remains an interesting phenomenon but cannot replace respiratory sampling for the diagnosis of acute COVID-19. Studies characterizing diagnostic performance suffer from heterogeneity, poor reporting practices, fundamental reference standard limitations, and lack of published studies in the Omicron era. Antigenemia appears very common in the first 2 weeks following symptom onset in hospitalized patients and disappears in nearly all patients by the end of the fourth week. Roles for antigenemia measurements in surveillance where blood specimens are already collected for other reasons, in the evaluation and treatment of immunocompromised patients presenting with complex syndromes, and as clues to the pathophysiology of sequelae of SARS-CoV-2 infection warrant ongoing investigation.

## Supplementary Material

ofae561_Supplementary_Data

## Supplementary Data

Supplementary materials are available at *Open Forum Infectious Diseases* online. Consisting of data provided by the authors to benefit the reader, the posted materials are not copyedited and are the sole responsibility of the authors, so questions or comments should be addressed to the corresponding author.
