# Supplementary material for: Diagnostic Utility of SARS-CoV-2 Nucleocapsid Antigenemia: A Meta-analysis
Source: Open Forum Infect Dis. 2024 Oct 2;11(10):ofae561. doi: 10.1093/ofid/ofae561 (PMC11487748; doi:10.1093/ofid/ofae561)
Supplement: ofae561_Supplementary_Data [file ofae561_supplementary_data.pdf]

Supplementary Information

Table S1. Pubmed search 2
Table S2. Scopus search 3
Table S3. Embase search 4
Table S4. Summary of source data. 6
Table S5. Cases included in variations on the acute COVID-19 meta-analysis. 7
Table S6. Principles for risk of bias and applicability judgements based on the QUADAS-2 framework. 8
Table S7. Studies meeting inclusion criteria without analyzable data 9
Table S9. Sensitivity analysis of assay subgroups with I^2 > 90% for the meta-analysis using 2.97 pg/mL cutoff. 11
Table S10. Risk of bias and applicability assessment for studies included in each meta-analysis. 12
Figure S1. PRISMA flow chart 13
Figure S2. Schematic of reference standards used in meta-analyses 14
Figure S3. Alternate meta-analyses of sensitivity and specificity for acute COVID-19 16
Figure S4. Alternate meta-analysis of nucleocapsid antigenemia as a diagnostic indicator of nasal swab viral load 18
Figure S5. Omicron era nucleocapsid antigenemia has a similar distribution to early pandemic antigenemia. 19
PRISMA 2020 Checklist 20
PRISMA 2020 for Abstracts Checklist 22
Data extraction template 24

Search number	Terms	Filters	Results 2/25/2023	Results 7/15/2023
8	#5 AND #6	from 2019/12/1 - 3000/12/12	1,836	2,079
7	#5 AND #6		2,156	2,399
6	"sars-cov-2"[Title/Abstract] OR "coronavirus"[Title/Abstract] OR "covid"[Title/Abstract]		341,114	374,391
5	#3 OR #4		152,352	154,153
4	antigenemia[Title/Abstract]		3,119	3,132
3	#1 AND #2		149,997	151,788
2	"nucleocapsid"[Title/Abstract] OR "n protein"[Title/Abstract] OR "antigen"[Title/Abstract]		511,597	518,955
1	"serum"[Title/Abstract] OR "blood"[Title/Abstract] OR "plasma"[Title/Abstract]		3,730,554	3,789,211

Table S1. Pubmed search

Search	Terms	Results 2/25/2023	Results 7/15/2023
9	(((TITLE-ABS ("serum" OR "blood" OR "plasma")) AND (TITLE-ABS ("nucleocapsid" OR "n protein" OR "antigen"))) OR (TITLE-ABS ("antigenemia"))) AND (TITLE-ABS ("sars-cov-2" OR "coronavirus" OR "covid")) AND PUBYEAR > 2018 AND PUBYEAR < 2024	2,182	2,483
8	(((TITLE-ABS ("serum" OR "blood" OR "plasma")) AND (TITLE-ABS ("nucleocapsid" OR "n protein" OR "antigen"))) OR (TITLE-ABS ("antigenemia"))) AND (TITLE-ABS ("sars-cov-2" OR "coronavirus" OR "covid"))	2,663	2,964
7	(((TITLE-ABS ("serum" OR "blood" OR "plasma")) AND (TITLE-ABS ("nucleocapsid" OR "n protein" OR "antigen"))) OR (TITLE-ABS ("antigenemia"))) AND (TITLE-ABS ("sars-cov-2" OR "coronavirus" OR "covid"))	2,663	2,964
6	TITLE-ABS ("sars-cov-2" OR "coronavirus" OR "covid")	457,447	515,394
5	((TITLE-ABS ("serum" OR "blood" OR "plasma")) AND (TITLE-ABS ("nucleocapsid" OR "n protein" OR "antigen"))) OR (TITLE-ABS ("antigenemia"))	234,768	237,201
4	TITLE-ABS ("antigenemia")	4,252	4,275
3	(TITLE-ABS ("serum" OR "blood" OR "plasma")) AND (TITLE-ABS ("nucleocapsid" OR "n protein" OR "antigen"))	231,828	234,248
2	TITLE-ABS ("nucleocapsid" OR "n protein" OR "antigen")	706,918	716,055
1	TITLE-ABS ("serum" OR "blood" OR "plasma")	5,020,091	5,102,642

Table S2. Scopus search

Search	Terms	Results 2/25/2023	Results 7/15/2023
#8	#5 AND #6 AND [01-12-2019]/sd	2,448	2,856
#7	#5 AND #6	2,753	3,161
#6	'sars-cov-2':ti OR 'coronavirus':ti OR 'covid':ti OR 'sars-cov-2':ab OR 'coronavirus':ab OR 'covid':ab	370,358	412,314
#5	#3 OR #4	202,879	205,911
#4	'antigenemia':ti OR 'antigenemia':ab	4,097	4,121
#3	#1 AND #2	199,745	202,756
#2	'nucleocapsid':ti OR 'n protein':ti OR 'antigen':ti OR 'nucleocapsid':ab OR 'n protein':ab OR 'antigen':ab	630,640	641,358
#1	'serum':ti OR 'blood':ti OR 'plasma':ti OR 'serum':ab OR 'blood':ab OR 'plasma':ab	4,972,209	5,067,056

Table S3. Embase search

Author	Year	Reference	Timing provided: Symptoms	Timing provided: Diagnosis	Nucleocapsid measurements provided	Source data included in least-restrictive meta-analysis as… Cases	Controls	Excluded from least-restrictive meta-analysis: Number	Reason
Blain	2022	[11]	No	No	56	56	0	0	NA
Damhorst	2023a	[12]	Yes	Yes	91	81	0	10	10 controls were not verified to be SC2 negative and were excluded.
Damhorst	2023b	[13]	Yes	Yes	54	30	0	24	Not all RT-PCR+ participants are considered acute COVID-19 in the manuscript. We have chosen to include only those within 14 days of symptom onset as acute cases in the meta-analysis. 24 participants had more than 14 days of symptoms.
Favresse	2022	[14]	Yes	Partial	243	179	0	64	A subset of patients had serial measurements. 64 of these measurements were from more than 14 days after symptom onset.
Ogata	2020	[15]	No	Partial	57	57	0	0	NA
Parraud	2023	[26]	No	Yes	59	42	0	17	Measurements up to 32 days after positive PCR were provided, those > 14 days were excluded.
Perna	2021	[16]	No	No	294	233	0	61	Not stated whether controls had negative SC2 testing, therefore excluded 61 controls.
Saini	2023	[17]	Partial	Partial	76	64	12	0	NA
Shan	2021	[10]	No	Yes	155	102	0	53	Excluded 53 measurements provided in supplement that were more than 14 days from positive PCR.
Sigal	2022	[18]	No	Yes	36	36	0	0	NA
Swank	2022	[19]	Partial	No	160	0	0	160	Study of PASC where most measurements occurred beyond 14 days of symptom onset. We did not include these data in the "least-restrictive" meta-analysis because the cohort was not explicitly defined as acute COVID-19 in the original study. However, we included the applicable patient-level data in the meta-analyses where cases were defined by time since symptom onset since the data were sufficient to satisfy the definition we established for these analyses.
Thudium	2021	[20]	Yes	No	914	341	467	106	Cases > 14 days since COVID-19 diagnosis were excluded due to a large number of late measurements.
Verkerke	2021	[21]	Yes	No	1221	429	0	792	Measurements beyond 14 days since symptom onset were excluded due to large number of late measurements.
Verkerke	2022	[22]	Yes	Yes	354	141	194	19	Excluded measurements are those from patients where paired Ct was available but timing from symptoms and from diagnosis were not available.
Veyrenche	2022	[23]	Yes	No	82	82	0	0	NA
Wang	2021	[24]	Yes	No	74	74	0	0	NA
Wick	2022	[25]	Yes	No	266	266	0	0	NA

Table S4. Summary of source data.

This table reports how patient-level measurements provided by study authors were handled in our analysis. While some studies provided a single measurement per patient/participant, others provided multiple (serial) measurements for the same patient including measurements weeks after COVID-19 symptom onset. To avoid the inclusion of these late measurements and biasing of the meta-analysis with excess negative measurements, we imposed parameters on which measurements could be included in the "least restrictive" meta-analysis (the meta-analysis that accepts the author's definition and does not universally restrict analysis to those with < 14 days of symptoms). Each study was handled on a case-by-case basis and an explanation is provided in the "Reason" column.
*Time since diagnosis was not specifically requested from corresponding authors.

			Cases included in meta-analysis variations							Controls in meta-analysis variations		
			Author’s index test cutoff			Index test cutoff 2.97-3.0 pg/mL				Index test cutoff		
First Author	Ref	Pub year	Author’s definition	≤ 14 days symptoms	≤ 7 days symptoms	Author’s definition	≤ 14 days symptoms	≤ 7 days symptoms	Data source	Per author	2.97-3.0 pg/mL	Data source
Ahava	[27]	2022	51	45	26	51	45	26	Table 1	--	--	--
Blain	[11]	2022	56	--	--	56	--	--	Source data	--	--	--
Chenane	[28]	2022	754	165	28	754	165	28	Abstract	--	--	--
Damhorst	[12]	2023	81	81	55	81	81	55	Source data	--	--	--
Damhorst	[13]	2023	30	30	22	30	30	22	Source data	--	--	--
Favresse	[14]	2022	179	179	114	179	179	114	Source data	--	--	--
Hingrat	[29]	2020	142	142	56	142	142	56	Figure 1	--	--	--
Jilg	[30]	2023	229	229	--	229	229	--	Abstract	--	--	--
Li	[31]	2020	50	--	--	--	--	--	Table 1	633	--	Table 4
Ogata	[15]	2020	57	--	--	57	--	--	Source data	17	17	Figure 1
Oueslati	[32]	2022	56	--	--	56	--	--	Text 3.3	42	42	Text 3.3
Parraud	[26]	2023	42	42	--	42	42	--	Source data	--	--	--
Perna	[16]	2021	233	--	--	233	--	--	Source data	--	--	--
Rogers	[33]	2022	2540	2540	--	2540	2540	--	Text p3	--	--	--
Saini	[17]	2023	64	--	--	64	--	--	Source data	12	12	Source data
Shan	[10]	2021	102	--	--	102	--	--	Source/ Supplement	--	--	--
Sigal	[18]	2022	36	--	--	36	--	--	Source data	43	--	Table 2
Su	[34]	2021	39	--	--	--	--	--	Text p2	50	--	Text p2
Sullivan	[35]	2023	638	--	--	638	--	--	Abstract	--	--	--
Swank	[19]	2022	--	--	--	--	6	--	Source data	--	--	--
Thudium	[20]	2021	341	--	--	341	--	--	Source data	467	467	Source data
Verkerke	[21]	2021	429	429	135	429	429	135	Source data	--	--	--
Verkerke	[22]	2022	141	141	69	141	141	69	Source data	194	194	Source data
Veyrenche	[23]	2022	82	58	16	82	58	16	Source data	--	--	--
Wang	[24]	2021	74	70	40	74	70	40	Source data	52	--	Text p5
Wick	[25]	2022	266	217	105	266	217	105	Source data	--	--	--
Yonker	[36]	2021	22	--	--	--	--	--	Figure 1	--	--	--
Zhang	[37]	2021	177	170	143	177	170	143	Figure 1	60	60	Table 1
Zhang	[38]	2022	140	127	59	--	--	--	Table 2	--	--	--

Table S5. Cases included in variations on the acute COVID-19 meta-analysis.

	Risk of bias characteristics		Applicability concern	
**Domain**	**Low**	**High**	**Low**	**High**
Sensitivity of antigenemia for acute COVID-19				
**Patient selection**	Consecutive or random enrollment	Convenience sampling, secondary specimen analysis, sampling from a broader cohort where not all participants had blood samples available	Study performed since the emergence of the omicron variant	Study performed prior to the emergence of the omicron variant
**Index test**	Cutoff value 2.97–3.0 pg/mL	Cutoff value other than 2.97–3.0 pg/mL	Commercially available assay	Lab-developed assays
**Reference standard**	Cases defined by positive respiratory RT-PCR *and* time from symptom onset	Timing not provided or based on time from positive RT-PCR only	--	--
**Flow and timing**	--	--	--	--
Diagnostic accuracy of antigenemia with respect to respiratory viral load				
**Patient selection**	Consecutive or random enrollment	Convenience sampling, secondary specimen analysis, sampling from a broader cohort where not all participants had blood samples available	Study performed since the emergence of the omicron variant	Study performed prior to the emergence of the omicron variant
**Index test**	Cutoff value 2.97–3.0 pg/mL	Cutoff value other than 2.97–3.0 pg/mL	Commercially available assay	Home-brew assays
**Reference standard**	One RT-PCR assay and sample type used	Multiple RT-PCR assays or sample types used	--	--
**Flow and timing**	RT-PCR performed within 24 hours of blood sample collection	*Not applicable*	--	--

Table S6. Principles for risk of bias and applicability judgements based on the QUADAS-2 framework.

First Author	Ref	Pub year	Location	Start date	End date	Assay
Brasen	[42]	2021	Denmark	3/30/2020	9/15/2020	Quanterix Simoa
Brasen	[43]	2023	Denmark	NR	NR	Quanterix Simoa
Epling	[44]	2023	Bethesda, MD	12/1/2021	5/31/2022	Quanterix Simoa
Kristiansen	[45]	2022	Denmark	11/1/2021	11/30/2021	Solsten ELISA
Lebedin	[46]	2021	Moscow, Russia	NR	NR	Hytest Ltd sandwich assay
Liang	[47]	2021	China	NR	NR	ELISA
Liu	[47]	2021	China	NR	NR	ELISA
Mathur	[48]	2022	San Francisco, CA	9/1/2020	4/13/2021	Quanterix Simoa
Matthay	[49]	2023	San Francisco, CA	6/1/2020	3/31/2021	Quanterix Simoa
Neumann	[50]	2021	NR	NR	NR	NR
Peluso	[51]	2023	USA	NR	NR	NR
Peluso	[52]	2023	USA	NR	NR	Quanterix Simoa
Rouka	[53]	2021	Larissa, Greece	NR	NR	Prognosis Biotech
Wu	[54]	2022	China	NR	NR	ELISA

Table S7. Studies meeting inclusion criteria without analyzable data (could not be extracted and source data not provided)

Study	Reference	Gene target(s)
Ahava 2022	[27]	Multiple
Hingrat 2020	[29]	E gene
Sigal 2022	[18]	N1 and N2 (averaged)
Wang 2021	[24]	Multiple
Zhang 2021	[37]	RdRp and N genes
Damhorst 2023b	[13]	N2
Favresse 2022A/C	[40]	N2 and E
Verkerke 2022	[22]	Multiple
Oueslati 2022	[32]	Multiple
Veyrenche 2022	[23]	E, N and RdRP

Table S8. Summary of gene targets used in the Ct value-based meta-analysis.

When not explicitly stated, the gene targets were determined based on package insert for the assay listed in each manuscript.

Sensitivity meta-analysis subgroup	Action	I^2 (95% CI)	Subgroup Sensitivity (95% CI)	Overall Sensitivity (95% CI)
**Author's definition (other assays)**	**No change**	**96% (94 – 98%)**	**0.885 (0.779 – 0.944)**	**0.793 (0.716 – 0.853)**
	Removal of Perna 2021	74% (41 – 89%)	0.919 (0.871 – 0.950)	0.803 (0.727 – 0.861)
**Author's definition (Quanterix simoa)**	**No change**	**97% (97 – 98%)**	**0.774 (0.663 – 0.856)**	**0.793 (0.716 – 0.853)**
	Removal of Saini 2023	97% (97 – 98%)	0.800 (0.705 – 0.870)	0.805 (0.734 – 0.862)
	Removal of Rogers 2022	95% (93 – 96%)	0.745 (0.638 – 0.829)	0.780 (0.702 – 0.843)
	Removal of Saini 2023 and Rogers 2022	94% (91 – 96%)	0.773 (0.683 – 0.844)	0.793 (0.720 – 0.851)
**<=14 days symptoms (Quanterix simoa)**	**No change**	**98% (97 – 98%)**	**0.773 (0.620 – 0.877)**	**0.830 (0.745 – 0.891)**
	Removal of Swank 2022	98% (97 – 98%)	0.810 (0.690 – 0.890)s	0.846 (0.776 – 0.898)

Table S9. Sensitivity analysis of assay subgroups with I^2 > 90% for the meta-analysis using 2.97 pg/mL cutoff.

The meta-sensitivity was recalculated for each subgroup with I^2 > 90% after removal of one or two apparent outliers. I^2 fell below 90% with the removal of a single outlier from the "other assays" subgroup for the "author's definition" sensitivity meta-analysis and raised the sensitivity estimate for that subgroup although 95% CI still overlapped substantially. In the "Quanterix simoa" subgroups, which included more studies, removal of outliers had a less pronounced effect on I^2 and sensitivity estimates remained overall similar. This exercise highlights the high heterogeneity of the source studies in this meta-analysis, but did not substantially alter overall sensitivity estimates – the largest change in overall sensitivity was +0.016.

			Risk of bias: Acute COVID-19 sensitivity			Risk of bias: Viral load diagnostic performance			Applicability concerns: Both meta-analyses	
**Author**	**Year**	**Ref**	**Patient selection**	**Index test**	**Reference standard**	**Patient selection**	**Index test**	**Reference standard**	**Patient selection**	**Index test**
Ahava	2022	[27]	☹	☺	☺	☹	☺	?	☹	?
Blain	2022	[11]	☺	☺	☹	--	--	--	☹	?
Chenane	2022	[28]	☹	☺	☺	--	--	--	☹	?
Damhorst	2023	[12]	☹	☺	☺	--	--	--	☹	☺
Damhorst	2023	[13]	☹	☺	☺	☹	☺	☺	☺	☺
Favresse	2022	[14]	☹	☺	☺	☹	☺	☹	?	☺
Hingrat	2020	[29]	☹	☺	☺	☹	☺	☹	☹	?
Jilg	2023	[30]	?	☺	☺	--	--	--	☹	☺
Li	2020	[31]	?	☹	☹	--	--	--	?	?
Ogata	2020	[15]	?	☺	☹	--	--	--	☹	☺
Oueslati	2022	[32]	?	☺	☹	?	☺	☹	☹	?
Parraud	2023	[26]	☹	☺	☺	--	--	--	☹	?
Perna	2021	[16]	?	☺	☹	--	--	--	?	☺
Rogers	2022	[33]	☺	☺	☺	--	--	--	☹	☺
Saini	2023	[17]	?	☺	☹	--	--	--	?	☺
Shan	2021	[10]	?	☺	☹	--	--	--	☹	☺
Sigal	2022	[18]	?	☺	☹	?	☺	☺	☹	☺
Su	2021	[34]	?	☹	☹	--	--	--	?	☺
Sullivan	2023	[35]	☹	☺	☹	--	--	--	☹	☺
Swank	2022	[19]	☹	☺	☺	--	--	--	?	☺
Thudium	2021	[20]	?	☺	☹	--	--	--	☹	☺
Verkerke	2021	[21]	☹	☺	☺	--	--	--	☹	☺
Verkerke	2022	[22]	☹	☺	☺	☹	☺	☹	☹	☺
Veyrenche	2022	[23]	?	☺	☺	?	☺	☺	☹	?
Wang	2021	[24]	☹	☺	☺	☹	☺	☹	☹	☺
Wick	2022	[25]	☹	☺	☺	--	--	--	☹	☺
Yonker	2021	[36]	?	☹	☹	--	--	--	?	☺
Zhang	2021	[37]	?	☺	☺	?	☺	☹	☹	?
Zhang	2022	[38]	☹	☹	☺	--	--	--	☹	?

Table S10. Risk of bias and applicability assessment for studies included in each meta-analysis.

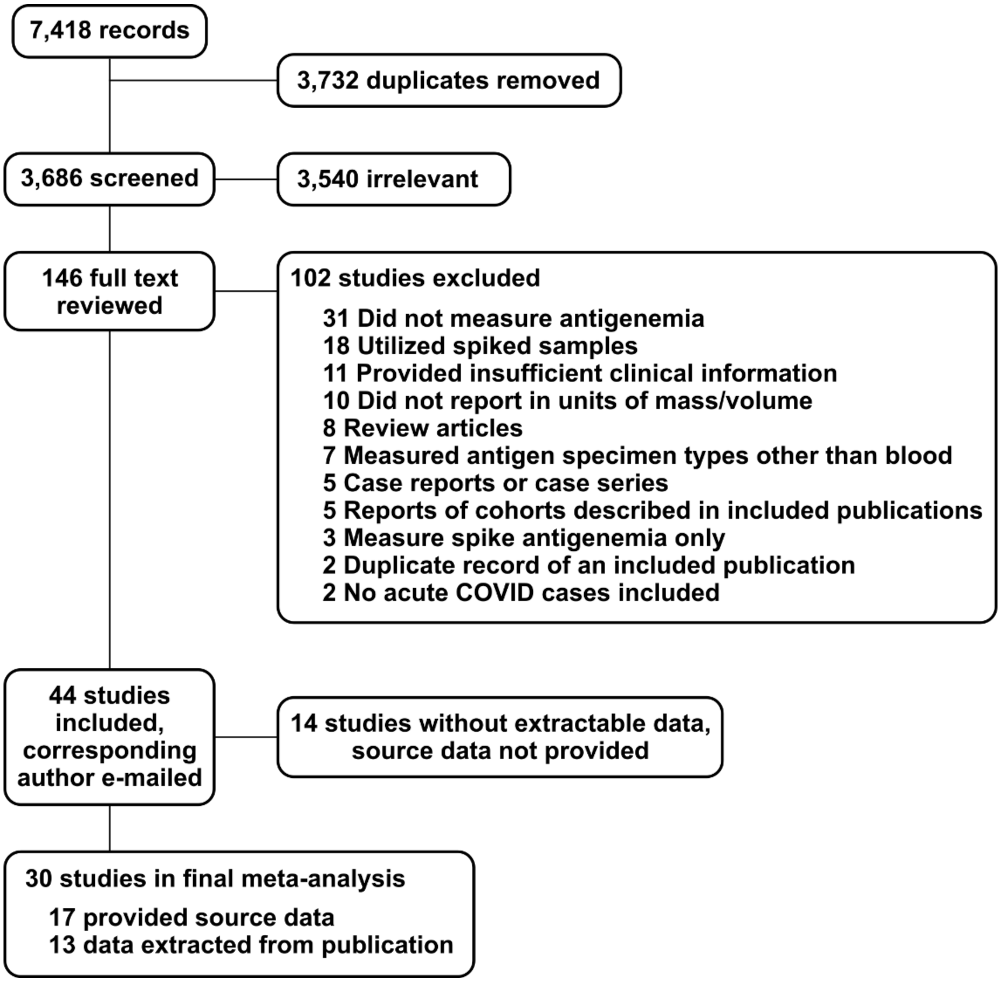

Figure S1. PRISMA flow chart

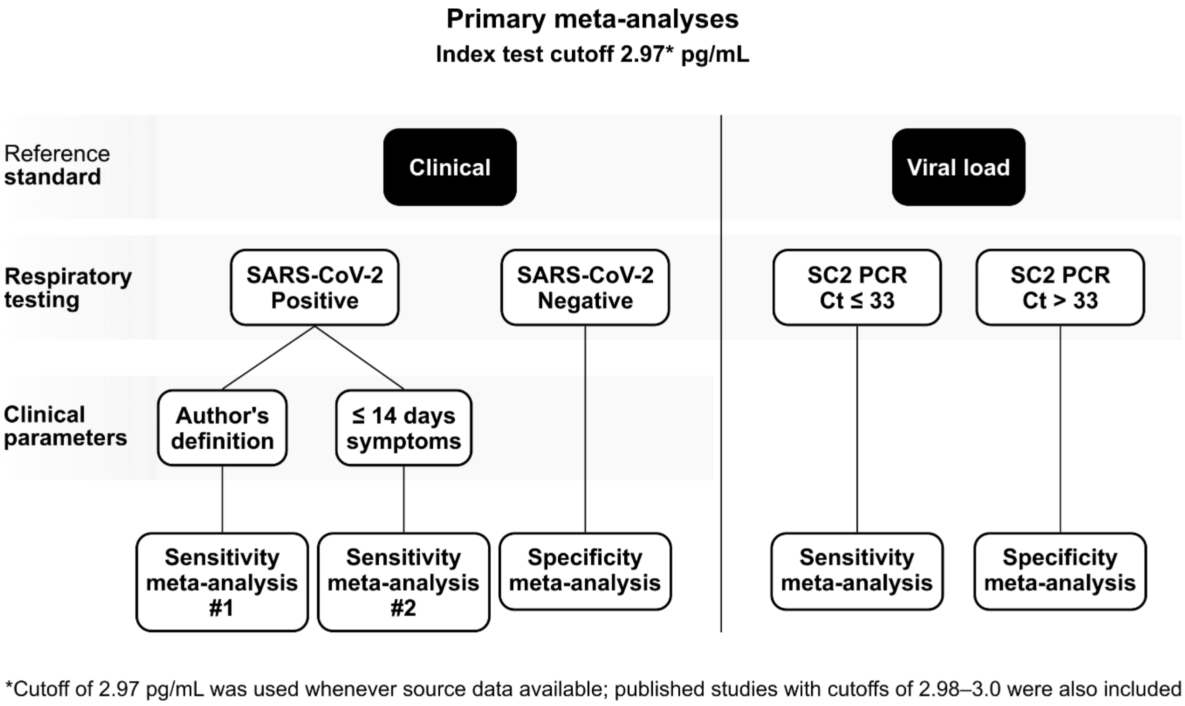

Figure S2. Schematic of reference standards used in meta-analyses

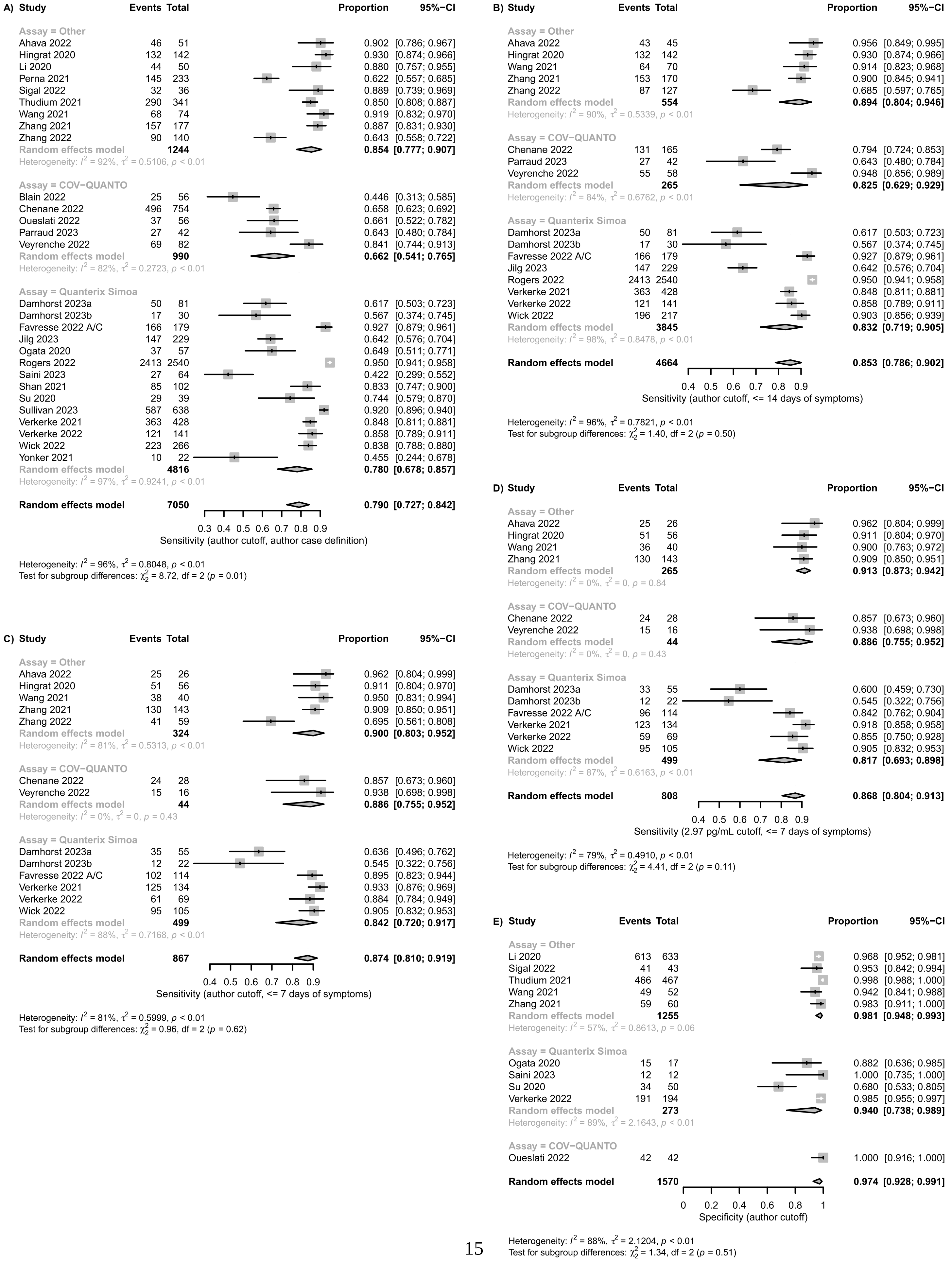
A) Study Events Total Proportion 95%−CI
Assay = Other
Ahava 2022 46 51 0.902 [0.786; 0.967]
Hingrat 2020 132 142 0.930 [0.874; 0.966]
Li 2020 44 50 0.880 [0.757; 0.955]
Perna 2021 145 233 0.622 [0.557; 0.685]
Sigal 2022 32 36 0.889 [0.739; 0.969]
Thudium 2021 290 341 0.850 [0.808; 0.887]
Wang 2021 68 74 0.919 [0.832; 0.970]
Zhang 2021 157 177 0.887 [0.831; 0.930]
Zhang 2022 90 140 0.643 [0.558; 0.722]
Random effects model 1244 0.854 [0.777; 0.907]
Heterogeneity: I^2 = 92%, τ^2 = 0.5106, p < 0.01
Assay = COV−QUANTO
Blain 2022 25 56 0.446 [0.313; 0.585]
Chenane 2022 496 754 0.658 [0.623; 0.692]
Oueslati 2022 37 56 0.661 [0.522; 0.782]
Parraud 2023 27 42 0.643 [0.480; 0.784]
Veyrenche 2022 69 82 0.841 [0.744; 0.913]
Random effects model 990 0.662 [0.541; 0.765]
Heterogeneity: I^2 = 82%, τ^2 = 0.2723, p < 0.01
Assay = Quanterix Simoa
Damhorst 2023a 50 81 0.617 [0.503; 0.723]
Damhorst 2023b 17 30 0.567 [0.374; 0.745]
Favresse 2022 A/C 166 179 0.927 [0.879; 0.961]
Jilg 2023 147 229 0.642 [0.576; 0.704]
Ogata 2020 37 57 0.649 [0.511; 0.771]
Rogers 2022 2413 2540 0.950 [0.941; 0.958]
Saini 2023 27 64 0.422 [0.299; 0.552]
Shan 2021 85 102 0.833 [0.747; 0.900]
Su 2020 29 39 0.744 [0.579; 0.870]
Sullivan 2023 587 638 0.920 [0.896; 0.940]
Verkerke 2021 363 428 0.848 [0.811; 0.881]
Verkerke 2022 121 141 0.858 [0.789; 0.911]
Wick 2022 223 266 0.838 [0.788; 0.880]
Yonker 2021 10 22 0.455 [0.244; 0.678]
Random effects model 4816 0.780 [0.678; 0.857]
Heterogeneity: I^2 = 97%, τ^2 = 0.9241, p < 0.01
Random effects model 7050 0.790 [0.727; 0.842]
0.3 0.4 0.5 0.6 0.7 0.8 0.9
Sensitivity (author cutoff, author case definition)
Heterogeneity: I^2 = 96%, τ^2 = 0.8048, p < 0.01
Test for subgroup differences: χ^2_2 = 8.72, df = 2 (p = 0.01)
B) Study Events Total Proportion 95%−CI
Assay = Other
Ahava 2022 43 45 0.956 [0.849; 0.995]
Hingrat 2020 132 142 0.930 [0.874; 0.966]
Wang 2021 64 70 0.914 [0.823; 0.968]
Zhang 2021 153 170 0.900 [0.845; 0.941]
Zhang 2022 87 127 0.685 [0.597; 0.765]
Random effects model 554 0.894 [0.804; 0.946]
Heterogeneity: I^2 = 90%, τ^2 = 0.5339, p < 0.01
Assay = COV−QUANTO
Chenane 2022 131 165 0.794 [0.724; 0.853]
Parraud 2023 27 42 0.643 [0.480; 0.784]
Veyrenche 2022 55 58 0.948 [0.856; 0.989]
Random effects model 265 0.825 [0.629; 0.929]
Heterogeneity: I^2 = 84%, τ^2 = 0.6762, p < 0.01
Assay = Quanterix Simoa
Damhorst 2023a 50 81 0.617 [0.503; 0.723]
Damhorst 2023b 17 30 0.567 [0.374; 0.745]
Favresse 2022 A/C 166 179 0.927 [0.879; 0.961]
Jilg 2023 147 229 0.642 [0.576; 0.704]
Rogers 2022 2413 2540 0.950 [0.941; 0.958]
Verkerke 2021 363 428 0.848 [0.811; 0.881]
Verkerke 2022 121 141 0.858 [0.789; 0.911]
Wick 2022 196 217 0.903 [0.856; 0.939]
Random effects model 3845 0.832 [0.719; 0.905]
Heterogeneity: I^2 = 98%, τ^2 = 0.8478, p < 0.01
Random effects model 4664 0.853 [0.786; 0.902]
0.4 0.5 0.6 0.7 0.8 0.9
Sensitivity (author cutoff, <= 14 days of symptoms)
Heterogeneity: I^2 = 96%, τ^2 = 0.7821, p < 0.01
Test for subgroup differences: χ^2_2 = 1.40, df = 2 (p = 0.50)
C) Study Events Total Proportion 95%−CI
Assay = Other
Ahava 2022 25 26 0.962 [0.804; 0.999]
Hingrat 2020 51 56 0.911 [0.804; 0.970]
Wang 2021 38 40 0.950 [0.831; 0.994]
Zhang 2021 130 143 0.909 [0.850; 0.951]
Zhang 2022 41 59 0.695 [0.561; 0.808]
Random effects model 324 0.900 [0.803; 0.952]
Heterogeneity: I^2 = 81%, τ^2 = 0.5313, p < 0.01
Assay = COV−QUANTO
Chenane 2022 24 28 0.857 [0.673; 0.960]
Veyrenche 2022 15 16 0.938 [0.698; 0.998]
Random effects model 44 0.886 [0.755; 0.952]
Heterogeneity: I^2 = 0%, τ^2 = 0, p = 0.43
Assay = Quanterix Simoa
Damhorst 2023a 35 55 0.636 [0.496; 0.762]
Damhorst 2023b 12 22 0.545 [0.322; 0.756]
Favresse 2022 A/C 102 114 0.895 [0.823; 0.944]
Verkerke 2021 125 134 0.933 [0.876; 0.969]
Verkerke 2022 61 69 0.884 [0.784; 0.949]
Wick 2022 95 105 0.905 [0.832; 0.953]
Random effects model 499 0.842 [0.720; 0.917]
Heterogeneity: I^2 = 88%, τ^2 = 0.7168, p < 0.01
Random effects model 867 0.874 [0.810; 0.919]
0.4 0.5 0.6 0.7 0.8 0.9
Sensitivity (author cutoff, <= 7 days of symptoms)
Heterogeneity: I^2 = 81%, τ^2 = 0.5999, p < 0.01
Test for subgroup differences: χ^2_2 = 0.96, df = 2 (p = 0.62)
D) Study Events Total Proportion 95%−CI
Assay = Other
Ahava 2022 25 26 0.962 [0.804; 0.999]
Hingrat 2020 51 56 0.911 [0.804; 0.970]
Wang 2021 36 40 0.900 [0.763; 0.972]
Zhang 2021 130 143 0.909 [0.850; 0.951]
Random effects model 265 0.913 [0.873; 0.942]
Heterogeneity: I^2 = 0%, τ^2 = 0, p = 0.84
Assay = COV−QUANTO
Chenane 2022 24 28 0.857 [0.673; 0.960]
Veyrenche 2022 15 16 0.938 [0.698; 0.998]
Random effects model 44 0.886 [0.755; 0.952]
Heterogeneity: I^2 = 0%, τ^2 = 0, p = 0.43
Assay = Quanterix Simoa
Damhorst 2023a 33 55 0.600 [0.459; 0.730]
Damhorst 2023b 12 22 0.545 [0.322; 0.756]
Favresse 2022 A/C 96 114 0.842 [0.762; 0.904]
Verkerke 2021 123 134 0.918 [0.858; 0.958]
Verkerke 2022 59 69 0.855 [0.750; 0.928]
Wick 2022 95 105 0.905 [0.832; 0.953]
Random effects model 499 0.817 [0.693; 0.898]
Heterogeneity: I^2 = 87%, τ^2 = 0.6163, p < 0.01
Random effects model 808 0.868 [0.804; 0.913]
0.4 0.5 0.6 0.7 0.8 0.9
Sensitivity (2.97 pg/mL cutoff, <= 7 days of symptoms)
Heterogeneity: I^2 = 79%, τ^2 = 0.4910, p < 0.01
Test for subgroup differences: χ^2_2 = 4.41, df = 2 (p = 0.11)
E) Study Events Total Proportion 95%−CI
Assay = Other
Li 2020 613 633 0.968 [0.952; 0.981]
Sigal 2022 41 43 0.953 [0.842; 0.994]
Thudium 2021 466 467 0.998 [0.988; 1.000]
Wang 2021 49 52 0.942 [0.841; 0.988]
Zhang 2021 59 60 0.983 [0.911; 1.000]
Random effects model 1255 0.981 [0.948; 0.993]
Heterogeneity: I^2 = 57%, τ^2 = 0.8613, p = 0.06
Assay = Quanterix Simoa
Ogata 2020 15 17 0.882 [0.636; 0.985]
Saini 2023 12 12 1.000 [0.735; 1.000]
Su 2020 34 50 0.680 [0.533; 0.805]
Verkerke 2022 191 194 0.985 [0.955; 0.997]
Random effects model 273 0.940 [0.738; 0.989]
Heterogeneity: I^2 = 89%, τ^2 = 2.1643, p < 0.01
Assay = COV−QUANTO
Oueslati 2022 42 42 1.000 [0.916; 1.000]
Random effects model 1570 0.974 [0.928; 0.991]
0 0.2 0.4 0.6 0.8 1
Specificity (author cutoff)
Heterogeneity: I^2 = 88%, τ^2 = 2.1204, p < 0.01
Test for subgroup differences: χ^2_2 = 1.34, df = 2 (p = 0.51)

Figure S3. Alternate meta-analyses of sensitivity and specificity for acute COVID-19. (A) Sensitivity where the least restrictive parameters where the author's index test cutoff and the author's definition of acute COVID-19 are accepted as published. Additional alternate sensitivity meta-analyses accepted the author's cutoff value for the index test but restricted the case definition to (B) patients with ≤ 14 days of symptoms or (C) patients with ≤ 7 days of symptoms. (D) Sensitivity using the restrictive index test cutoff only for patients with ≤ 7 days of symptoms. (E) Alternate specificity meta-analysis accepting the author's index test cutoff value.

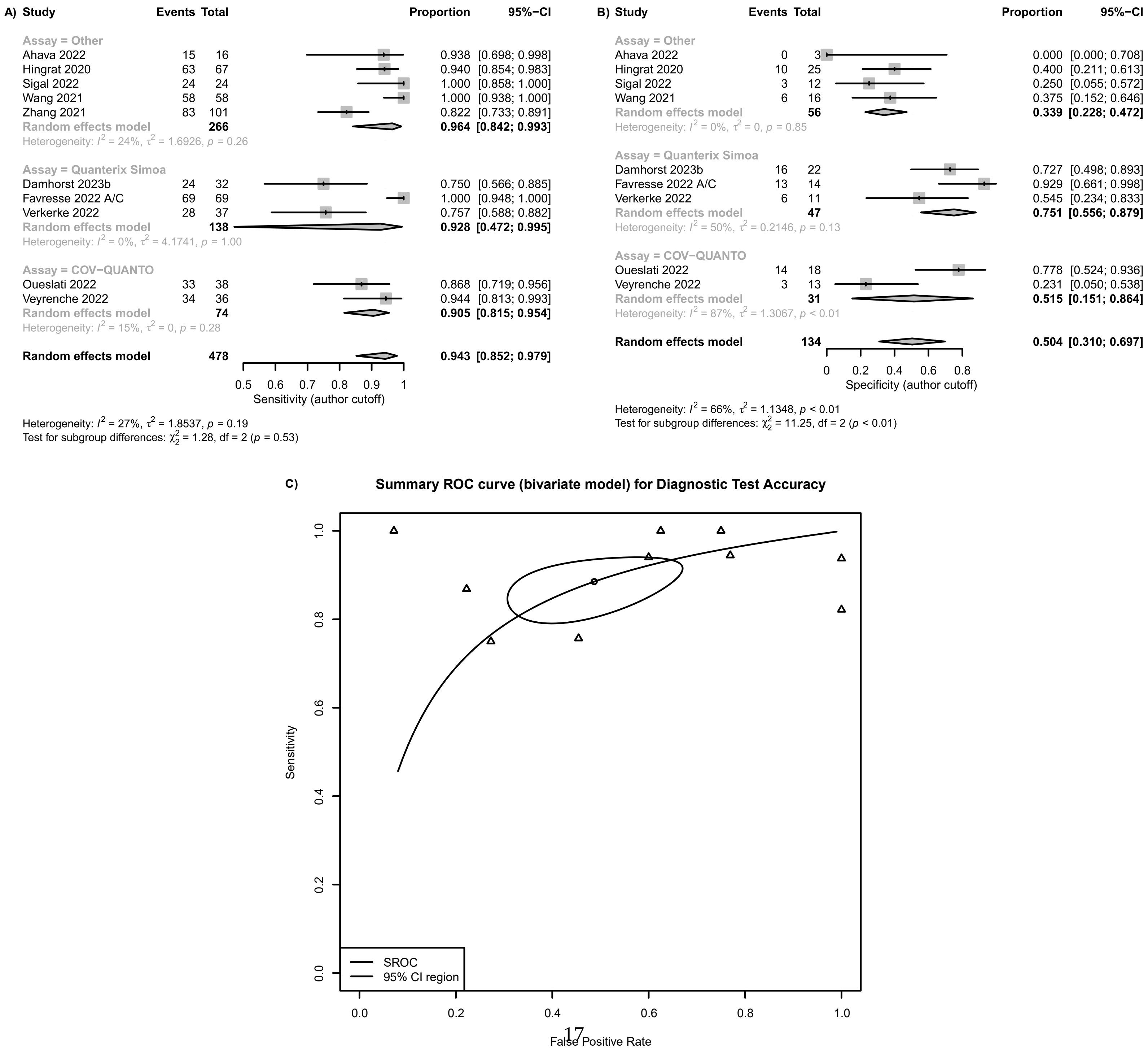

A) Study Events Total Proportion 95%−CI
Assay = Other
Ahava 2022 15 16 0.938 [0.698; 0.998]
Hingrat 2020 63 67 0.940 [0.854; 0.983]
Sigal 2022 24 24 1.000 [0.858; 1.000]
Wang 2021 58 58 1.000 [0.938; 1.000]
Zhang 2021 83 101 0.822 [0.733; 0.891]
Random effects model 266 0.964 [0.842; 0.993]
Heterogeneity: I^2 = 24%, τ^2 = 1.6926, p = 0.26
Assay = Quanterix Simoa
Damhorst 2023b 24 32 0.750 [0.566; 0.885]
Favresse 2022 A/C 69 69 1.000 [0.948; 1.000]
Verkerke 2022 28 37 0.757 [0.588; 0.882]
Random effects model 138 0.928 [0.472; 0.995]
Heterogeneity: I^2 = 0%, τ^2 = 4.1741, p = 1.00
Assay = COV−QUANTO
Oueslati 2022 33 38 0.868 [0.719; 0.956]
Veyrenche 2022 34 36 0.944 [0.813; 0.993]
Random effects model 74 0.905 [0.815; 0.954]
Heterogeneity: I^2 = 15%, τ^2 = 0, p = 0.28
Random effects model 478 0.943 [0.852; 0.979]
0.5 0.6 0.7 0.8 0.9 1
Sensitivity (author cutoff)
Heterogeneity: I^2 = 27%, τ^2 = 1.8537, p = 0.19
Test for subgroup differences: χ^2_2 = 1.28, df = 2 (p = 0.53)
B) Study Events Total Proportion 95%−CI
Assay = Other
Ahava 2022 0 3 0.000 [0.000; 0.708]
Hingrat 2020 10 25 0.400 [0.211; 0.613]
Sigal 2022 3 12 0.250 [0.055; 0.572]
Wang 2021 6 16 0.375 [0.152; 0.646]
Random effects model 56 0.339 [0.228; 0.472]
Heterogeneity: I^2 = 0%, τ^2 = 0, p = 0.85
Assay = Quanterix Simoa
Damhorst 2023b 16 22 0.727 [0.498; 0.893]
Favresse 2022 A/C 13 14 0.929 [0.661; 0.998]
Verkerke 2022 6 11 0.545 [0.234; 0.833]
Random effects model 47 0.751 [0.556; 0.879]
Heterogeneity: I^2 = 50%, τ^2 = 0.2146, p = 0.13
Assay = COV−QUANTO
Oueslati 2022 14 18 0.778 [0.524; 0.936]
Veyrenche 2022 3 13 0.231 [0.050; 0.538]
Random effects model 31 0.515 [0.151; 0.864]
Heterogeneity: I^2 = 87%, τ^2 = 1.3067, p < 0.01
Random effects model 134 0.504 [0.310; 0.697]
0 0.2 0.4 0.6 0.8
Specificity (author cutoff)
Heterogeneity: I^2 = 66%, τ^2 = 1.1348, p < 0.01
Test for subgroup differences: χ^2_2 = 11.25, df = 2 (p < 0.01)
C)
Summary ROC curve (bivariate model) for Diagnostic Test Accuracy
Sensitivity
0.0 0.2 0.4 0.6 0.8 1.0
False Positive Rate
0.0 0.2 0.4 0.6 0.8 1.0
SROC
95% CI region

Figure S4. Alternate meta-analysis of nucleocapsid antigenemia as a diagnostic indicator of nasal swab viral load using the author’s index test cutoff value. Univariate models for (A) sensitivity and (B) specificity, and (C) summary ROC curve using a bivariate model.

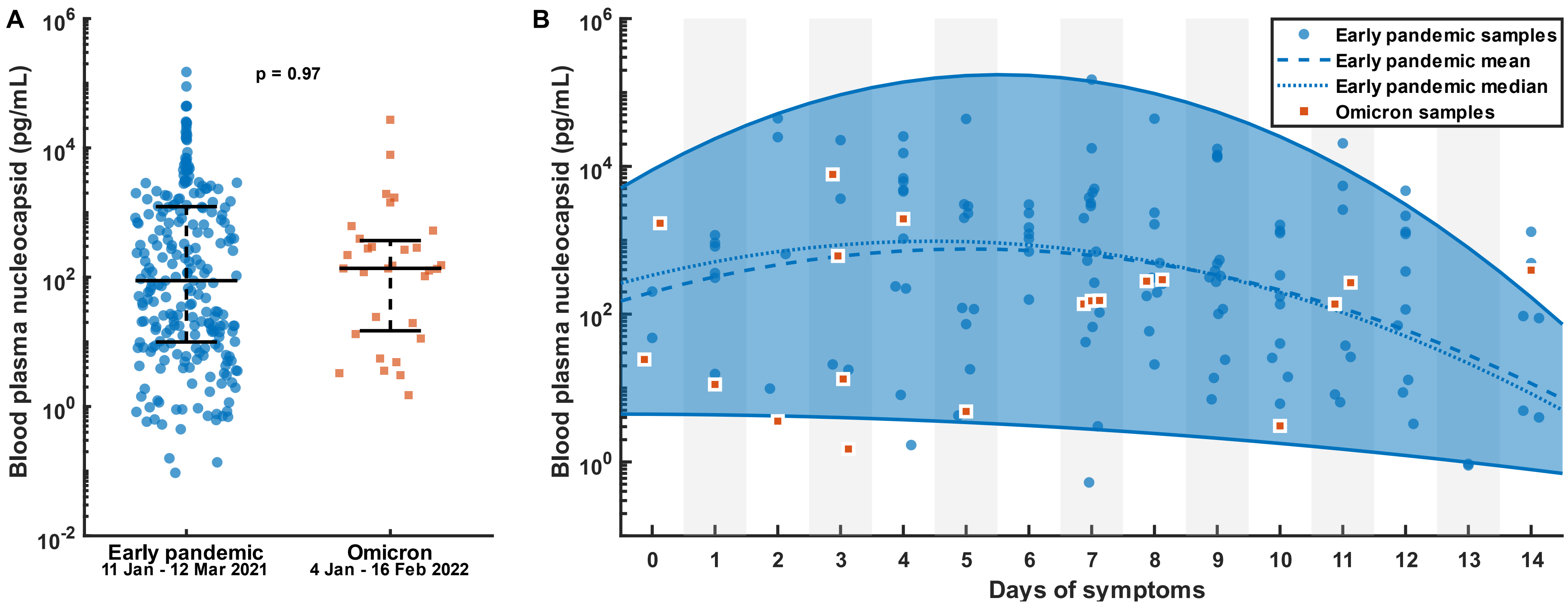

Figure S5. Omicron era nucleocapsid antigenemia has a similar distribution to early pandemic antigenemia. (A) Plasma nucleocapsid levels measured during the early pandemic (N = 244, 88.4 pg/mL [IQR 9.9-1235.5]) compared to the Omicron era (N =31, 137.1 pg/mL [IQR 14.8-368.5]) exhibited similar median values and were not significantly different by rank sum test. (B) Data binned by days since symptom onset. Mean, median and standard deviation (SD) of the log antigenemia level were calculated from early pandemic data in each bin. Quadratic regressions were calculated to fit mean, median and mean+/-1.96*SD versus time which defined the boundaries of a 95% distribution interval. 16 of 19 (84%) of Omicron era measurements fell within the 95% distribution estimate suggesting they follow a similar distribution pattern but may fall lower than the mean and median of early pandemic levels.

PRISMA 2020 Checklist

Section and Topic	Item #	Checklist item	Location where item is reported
**TITLE**			
Title	1	Identify the report as a systematic review.	Title
**ABSTRACT**			
Abstract	2	See the PRISMA 2020 for Abstracts checklist.	Supplement
**INTRODUCTION**			
Rationale	3	Describe the rationale for the review in the context of existing knowledge.	Introduction
Objectives	4	Provide an explicit statement of the objective(s) or question(s) the review addresses.	Introduction
**METHODS**			
Eligibility criteria	5	Specify the inclusion and exclusion criteria for the review and how studies were grouped for the syntheses.	Methods
Information sources	6	Specify all databases, registers, websites, organisations, reference lists and other sources searched or consulted to identify studies. Specify the date when each source was last searched or consulted.	Methods
Search strategy	7	Present the full search strategies for all databases, registers and websites, including any filters and limits used.	Supplement
Selection process	8	Specify the methods used to decide whether a study met the inclusion criteria of the review, including how many reviewers screened each record and each report retrieved, whether they worked independently, and if applicable, details of automation tools used in the process.	Methods
Data collection process	9	Specify the methods used to collect data from reports, including how many reviewers collected data from each report, whether they worked independently, any processes for obtaining or confirming data from study investigators, and if applicable, details of automation tools used in the process.	Methods
Data items	10a	List and define all outcomes for which data were sought. Specify whether all results that were compatible with each outcome domain in each study were sought (e.g. for all measures, time points, analyses), and if not, the methods used to decide which results to collect.	Methods
	10b	List and define all other variables for which data were sought (e.g. participant and intervention characteristics, funding sources). Describe any assumptions made about any missing or unclear information.	Methods
Study risk of bias assessment	11	Specify the methods used to assess risk of bias in the included studies, including details of the tool(s) used, how many reviewers assessed each study and whether they worked independently, and if applicable, details of automation tools used in the process.	Methods
Effect measures	12	Specify for each outcome the effect measure(s) (e.g. risk ratio, mean difference) used in the synthesis or presentation of results.	Methods
Synthesis methods	13a	Describe the processes used to decide which studies were eligible for each synthesis (e.g. tabulating the study intervention characteristics and comparing against the planned groups for each synthesis (item #5)).	Methods
	13b	Describe any methods required to prepare the data for presentation or synthesis, such as handling of missing summary statistics, or data conversions.	Methods
	13c	Describe any methods used to tabulate or visually display results of individual studies and syntheses.	Methods
	13d	Describe any methods used to synthesize results and provide a rationale for the choice(s). If meta-analysis was performed, describe the model(s), method(s) to identify the presence and extent of statistical heterogeneity, and software package(s) used.	Methods
	13e	Describe any methods used to explore possible causes of heterogeneity among study results (e.g. subgroup analysis, meta-regression).	N/A
	13f	Describe any sensitivity analyses conducted to assess robustness of the synthesized results.	N/A

Section and Topic	Item #	Checklist item	Location where item is reported
Reporting bias assessment	14	Describe any methods used to assess risk of bias due to missing results in a synthesis (arising from reporting biases).	Methods
Certainty assessment	15	Describe any methods used to assess certainty (or confidence) in the body of evidence for an outcome.	Methods
**RESULTS**			
Study selection	16a	Describe the results of the search and selection process, from the number of records identified in the search to the number of studies included in the review, ideally using a flow diagram.	Results
	16b	Cite studies that might appear to meet the inclusion criteria, but which were excluded, and explain why they were excluded.	Results
Study characteristics	17	Cite each included study and present its characteristics.	Table 1
Risk of bias in studies	18	Present assessments of risk of bias for each included study.	Table 2
Results of individual studies	19	For all outcomes, present, for each study: (a) summary statistics for each group (where appropriate) and (b) an effect estimate and its precision (e.g. confidence/credible interval), ideally using structured tables or plots.	Figs 2 and 3
Results of syntheses	20a	For each synthesis, briefly summarise the characteristics and risk of bias among contributing studies.	Table 2, Table S6
	20b	Present results of all statistical syntheses conducted. If meta-analysis was done, present for each the summary estimate and its precision (e.g. confidence/credible interval) and measures of statistical heterogeneity. If comparing groups, describe the direction of the effect.	Figs 2 and 3
	20c	Present results of all investigations of possible causes of heterogeneity among study results.	N/A
	20d	Present results of all sensitivity analyses conducted to assess the robustness of the synthesized results.	N/A
Reporting biases	21	Present assessments of risk of bias due to missing results (arising from reporting biases) for each synthesis assessed.	N/A
Certainty of evidence	22	Present assessments of certainty (or confidence) in the body of evidence for each outcome assessed.	Figs 2 and 3
**DISCUSSION**			
Discussion	23a	Provide a general interpretation of the results in the context of other evidence.	Discussion
	23b	Discuss any limitations of the evidence included in the review.	Discussion
	23c	Discuss any limitations of the review processes used.	Discussion
	23d	Discuss implications of the results for practice, policy, and future research.	Discussion
**OTHER INFORMATION**			
Registration and protocol	24a	Provide registration information for the review, including register name and registration number, or state that the review was not registered.	Methods
	24b	Indicate where the review protocol can be accessed, or state that a protocol was not prepared.	Methods
	24c	Describe and explain any amendments to information provided at registration or in the protocol.	N/A
Support	25	Describe sources of financial or non-financial support for the review, and the role of the funders or sponsors in the review.	Methods
Competing	26	Declare any competing interests of review authors.	Acknowled-

Section and Topic	Item #	Checklist item	Location where item is reported
interests			gements
Availability of data, code and other materials	27	Report which of the following are publicly available and where they can be found: template data collection forms; data extracted from included studies; data used for all analyses; analytic code; any other materials used in the review.	Acknowled-gements

From: Page MJ, McKenzie JE, Bossuyt PM, Boutron I, Hoffmann TC, Mulrow CD, et al. The PRISMA 2020 statement: an updated guideline for reporting systematic reviews. BMJ 2021;372:n71. doi: 10.1136/bmj.n71

For more information, visit: http://www.prisma-statement.org/

PRISMA 2020 for Abstracts Checklist

Section and Topic	Item #	Checklist item	Reported (Yes/No)
**TITLE**			
Title	1	Identify the report as a systematic review.	Yes
**BACKGROUND**			
Objectives	2	Provide an explicit statement of the main objective(s) or question(s) the review addresses.	Yes
**METHODS**			
Eligibility criteria	3	Specify the inclusion and exclusion criteria for the review.	Yes
Information sources	4	Specify the information sources (e.g. databases, registers) used to identify studies and the date when each was last searched.	Yes
Risk of bias	5	Specify the methods used to assess risk of bias in the included studies.	Yes
Synthesis of results	6	Specify the methods used to present and synthesise results.	Yes
**RESULTS**			
Included studies	7	Give the total number of included studies and participants and summarise relevant characteristics of studies.	Yes
Synthesis of results	8	Present results for main outcomes, preferably indicating the number of included studies and participants for each. If meta-analysis was done, report the summary estimate and confidence/credible interval. If comparing groups, indicate the direction of the effect (i.e. which group is favoured).	Yes
**DISCUSSION**			
Limitations of evidence	9	Provide a brief summary of the limitations of the evidence included in the review (e.g. study risk of bias, inconsistency and imprecision).	Yes
Interpretation	10	Provide a general interpretation of the results and important implications.	Yes
**OTHER**			
Funding	11	Specify the primary source of funding for the review.	Yes
Registration	12	Provide the register name and registration number.	No

From: Page MJ, McKenzie JE, Bossuyt PM, Boutron I, Hoffmann TC, Mulrow CD, et al. The PRISMA 2020 statement: an updated guideline for reporting systematic reviews. BMJ 2021;372:n71. doi: 10.1136/bmj.n71

For more information, visit: http://www.prisma-statement.org/

Data extraction template

		mm/dd/yyyy	*mm/dd/yyyy*		*#*	**Definition of a case**				*y/n*
**Yr**	**Location**	**Date_start**	**Date_end**	**Assay**	**Cutoff_pg_ml**	**RT-PCR+**	**Days_from_pos**	**Days_from_sympt**	**Comment**	**By_sympt_day**

	# total	*# pos*	*# neg*		*# total*	*# pos*	*# neg*	*# total*	*# pos*	*# neg*	*Case source*
**Cutoff_d1**	**Cases_7d**	**Ag_pos_7d**	**Ag_neg_7d**	**Cutoff_d2**	**Cases_14d**	**Ag_pos_14d**	**Ag_neg_14d**	**Cases**	**Ag_pos**	**Ag_neg**	

**Define control**		*# total*	*# pos*	*# neg*	*# total*	*# pos*	*# neg*
**RT-PCR-**	**Comment**	**Controls**	**Ag_pos_ctrl**	**Ag_neg_ctrl**	**Pre-pandemic**	**Ag_pos_PP**	**Ag_neg_PP**

y/n	*y/n*	*# total*	*# pos*	*# neg*	*# total*	*# pos*	*# neg*	*Ct source*
**Paired_sample_24**	**Ct_33**	**Pts_LT33**	**Ag_pos_LT33**	**Ag_neg_LT33**	**Pts_GT33**	**Ag_pos_GT33**	**Ag_neg_GT33**	
